# A proapoptotic effect of valproic acid on progenitors of embryonic stem cell-derived glutamatergic neurons

**DOI:** 10.1038/cddis.2013.205

**Published:** 2013-06-20

**Authors:** R Fujiki, A Sato, M Fujitani, T Yamashita

**Affiliations:** 1 Department of Molecular Neuroscience, Graduate School of Medicine, Osaka University, 2-2 Yamadaoka, Suita, Osaka 565-0871, Japan; 2 JST, CREST, 5, Sanbancho, Chiyoda-ku, Tokyo 102-0075, Japan

**Keywords:** ES-derived glutamatergic neuron, neural progenitor cell, valproic acid, apoptosis, histone acetylation

## Abstract

Valproic acid (VPA) is a branched-chain saturated fatty acid with a long history of clinical use as an antiepileptic drug (AED). VPA is also known to inhibit histone deacetylases (HDACs) and to cause diverse effects on neural progenitor cells (NPCs) and neurons. Although the neuroprotective or neurodestructive effects of VPA have been investigated in heterogeneous cell populations, in this study, we used homogeneous populations of NPCs and glutamatergic cortical pyramidal neurons, which were differentiated from embryonic stem (ES) cells. At therapeutic concentrations, VPA had a proapoptotic effect on ES cell-derived NPCs of glutamatergic neurons, but not on their progeny. This effect of VPA most likely occurred through the inhibition of HDACs, because similar phenotypes were observed following treatment with other HDAC inhibitors (HDACis) such as trichostatin A and sodium butyrate. The proapoptotic phenotype was not observed when cells were exposed to a structural analog of VPA, valpromide (VPM), which has the same antiepileptic effect as VPA, but does not inhibit HDACs. Western blotting confirmed that treatment with HDACis, but not VPM, significantly increased the levels of histone H3 acetylation in NPCs. HDACi treatments did not affect the survival of neurons, although the acetylation levels were increased to a limited extent. These results, which are based on a homogeneous culture system, suggest that VPA inhibits HDAC activity and induces the apoptosis of NPCs that are fated to differentiate into glutamatergic neurons. The dose-dependent effects of VPA both on apoptosis and hyperacetylation of histone H3 in NPCs supported this notion. These cell type- and differentiation stage-specific effects of VPA imply that dysfunction of HDACs during pregnancy significantly increase the risk of congenital malformations associated with VPA administration.

Valproic acid (VPA) is a branched-chain saturated fatty acid with a long history of clinical use as an antiepileptic drug (AED); it is also widely used for the treatment of mood disorders.^1^ Although the mechanism of action of VPA is not fully understood, it may reduce neuronal activity in the human brain by blocking sodium and calcium channels and enhancing *γ*-aminobutyric acid (GABA) function by inhibiting GABA transaminase.^2, 3^ VPA also directly inhibits histone deacetylases (HDACs) and relieves HDAC-dependent transcriptional repression by inducing hyperacetylated histones.^4^ VPA is classified as a broad-spectrum inhibitor against class I (HDAC1, HDAC2, HDAC3 and HDAC8) and class IIb (HDAC6 and HDAC10) HDAC families.^5^ Recently, it has been demonstrated that VPA possesses antitumor activity by inhibiting tumor proliferation and angiogenesis, and by inducing tumor senescence, cell death or differentiation in various tumor models both *in vitro* and *in vivo*.^6, 7, 8^ The exact antitumor mechanisms of VPA remain unclear, but HDAC inhibition, extracellular regulated kinase (ERK) activation, protein kinase C (PKC) inhibition, Wnt (wingless)-signaling activation (inhibition of glycogen synthase kinase-3*β* (GSK-3*β*)), peroxisome proliferator-activated receptor activation, proteasomal degradation of HDAC and DNA demethylation participate in its antitumor actions.^8, 9, 10, 11, 12, 13, 14^ Among these mechanisms, hyperacetylation of histones, as a result of HDAC inhibition, seems to be the most important one. VPA is also known as a human teratogen.^15, 16^ Maternal ingestion of medication during pregnancy is associated with significantly increased risks for major congenital malformations, especially spina bifida.^17^ Several studies have proposed that HDAC inhibition by VPA is closely related to teratogenesis in vertebrate embryos.^4, 18, 19^ A growing body of evidence suggests that VPA has neuroprotective and neurotrophic effects on neural progenitor cells (NPCs) and neurons, apart from its function as an AED. It promotes neuroplasticity, neurogenesis and cell survival. However, the precise molecular mechanisms of these effects have not been fully understood. Recent studies suggest that HDAC inhibition, PKC inhibition, inhibition of GSK-3*β* via Wnt-mediated signaling, ERK activation and phosphatidylinositol-3 kinase-protein kinase A activation by brain-derived neurotrophic factor, all mediate the actions of VPA. Furthermore, many of these pathways are interconnected.^20, 21, 22, 23, 24, 25, 26, 27, 28^ Despite numerous reports citing evidence of the neuroprotective effects of VPA, there are also some contradictory data, which show proapoptotic effects of VPA and other HDAC inhibitors (HDACis) on NPCs and neurons.^29, 30, 31, 32^ Considering the antitumor activity and teratogenic effects of VPA in embryos, one would predict, from the standpoint of division potential, that VPA might possess proapoptotic effects on NPCs.

To date, the effects of VPA on NPCs or neurons have been analyzed *in vivo* or by using heterogeneous primary cell cultures. Therefore, the different cell types (glutamatergic, AChergic, GABAergic or DAergic) and/or differentiation stages characteristic of the model systems used in earlier studies might lead to a misinterpretation of the effects of VPA. In this study, we have utilized a more homogeneous population of cells by using a sophisticated *in vitro* differentiation system in order to minimize problems associated with the use of mixed-cell populations. Bibel *et al.*^33, 34^ recently established a retinoic acid-treated embryoid body-based differentiation protocol that promotes the generation of highly homogeneous glutamatergic cortical pyramidal neurons from embryonic stem (ES) cells. The purity of this population is known to reach 90–95%, which is the highest efficiency of differentiation to glutamatergic neurons ever reported.^35, 36^ Furthermore, our approach led to substantial gains in neuronal survival.^37^ VPA did not have a proapoptotic effect on ES cell-derived glutamatergic neurons, but did demonstrate proapoptotic effects on NPCs at therapeutic concentrations (0.3–0.7 mM).^4,11^ The effect of VPA was most likely through the inhibition of HDACs, because similar phenotypes were observed following treatment with either of the two other HDACis, trichostatin A (TSA) and sodium butyrate (NaB). These phenotypes were not observed after treatment with valpromide (VPM), which is a structural analog of VPA having the same antiepileptic effect as VPA but lacking the HDACi activity. The levels of histone H3 acetylation was indeed increased in NPCs by HDACis, but not with VPM. HDACi treatments did not affect the survival of neurons, although the acetylation levels were increased to a moderate degree. Taken together with previous reports,^25, 27^ these data suggest that VPA, primarily by the inhibition of HDACs, suppresses apoptosis and induces neuronal differentiation of heterogeneous NPCs, but contrarily induces apoptosis of homogeneous NPCs that are fated to differentiate into glutamatergic neurons. We also observed that VPA had dose-dependent effects both on apoptosis and hyperacetylation of histone H3 in NPCs, and this also strongly indicates a correlation between hyperacetylation of histones and apoptosis.

## Results

### VPA, TSA and NaB, but not VPM, have proapoptotic effects on NPCs of ES cell-derived glutamatergic neurons

**Figure 1:**
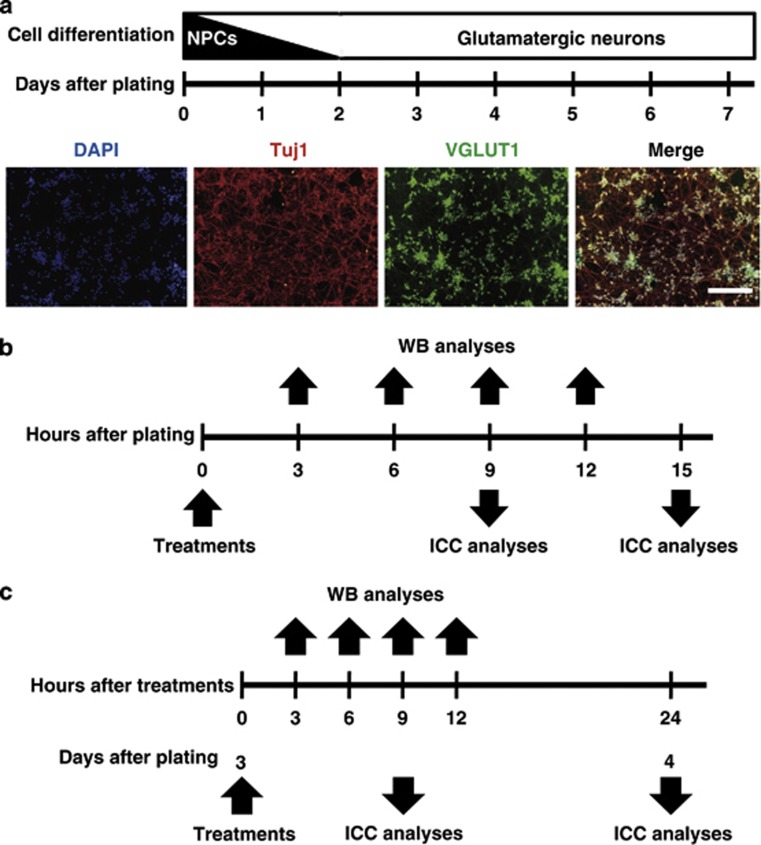
Schematic illustrations. (**a**) The upper scheme demonstrates how NPCs differentiate into glutamatergic neurons in our culture system. The lower panels are representative images of neurons 7 days after plating in our culture system. Over 97% neurons are positive in VGLUT1 (a glutamatergic neuronal marker), based on immunofluorescence analysis with the neuronal marker Tuj1 (red) and VGLUT1 (green). Nuclei were counterstained with DAPI (blue). Scale bar: 200 *μ*m. (**b**) Scheme shows the time schedule of ICC and WB analyses after treatment of NPCs with one of the four reagents (TSA, NaB, VPA or VPM) or their vehicle controls at day 0. (**c**) Schema shows the time schedule of ICC and WB analyses after treating glutamatergic neurons with one of the four reagents or their vehicle control at day 3

**Figure 2:**
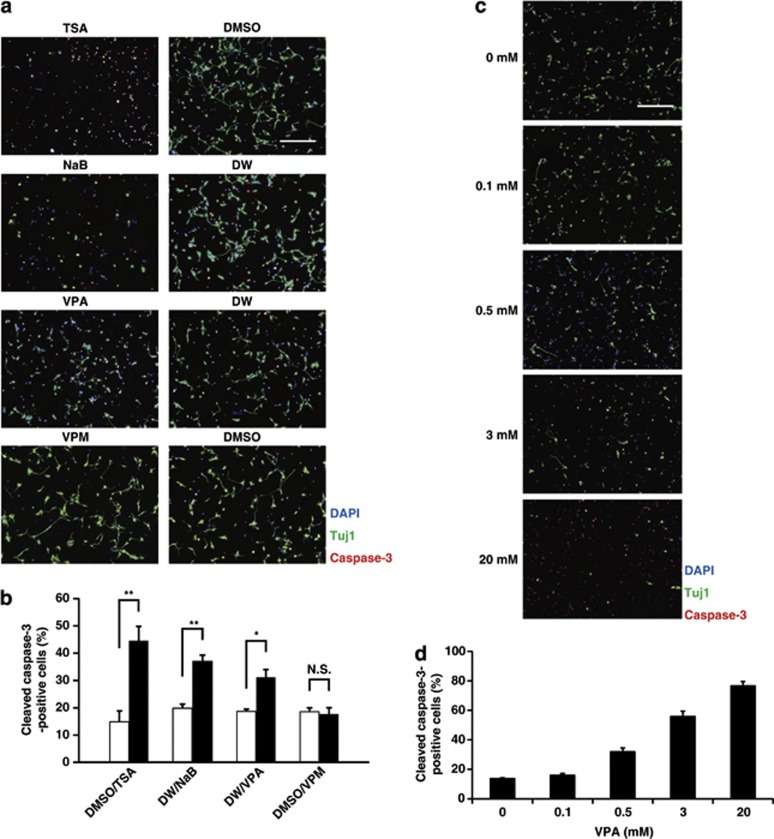
VPA has a proapoptotic effect on NPCs of ES cell-derived glutamatergic neurons, which is correlated with inhibition of HDACs. We treated our NPCs with one of the four reagents or their vehicle control at day 0, and investigated caspase-3 activation by using ICC 15 h later. We also treated NPCs at day 0 with different concentrations of VPA, and investigated caspase-3 activation by using ICC 9 h later. (**a**) Representative images of NPCs after a 15-h exposure to one of the four reagents or their vehicle control at day 0. Neurons were analyzed by immunofluorescently labeling cells with the neuronal marker Tuj1 (green) and the apoptosis marker cleaved caspase-3 (red). Nuclei were counterstained with DAPI (blue). Scale bar: 200 *μ*m. (**b**) Quantification of the percentage of the cleaved caspase-3-positive neural cells (cleaved capase-3^+^ cells among all Tuj1^+^ neurons) after a 15-h exposure to one of the four reagents or their vehicle control at day 0. We obtained four representative images, and counted all of the cells in those images (>200 cells) for one experiment, unless otherwise indicated. Note that VPA, TSA and NaB, but not VPM, had proapoptotic effects on NPCs of ES cell-derived glutamatergic neurons. Values represent the mean±S.E.M. of 5–7 separate experiments. TSA and its control (*n*=5); NaB and its control (*n*=6); VPA, VPM and their own controls (*n*=7). *P<0.05; **P<0.01; NS, not significant by one-way ANOVA followed by Tukey–Kramer's *post hoc* test. (**c**) Representative images of NPCs after a 9-h exposure to indicated concentrations of VPA at day 0. Neurons were analyzed by immunofluorescently labeling cells with the neuronal marker Tuj1 (green) and the apoptosis marker cleaved caspase-3 (red). Nuclei were counterstained with DAPI (blue). Scale bar: 200 *μ*m. (**d**) Quantification of the cleaved caspase-3-positive neural cells (cleaved capase-3^+^ cells among all Tuj1^+^ neurons) after a 9-h exposure to VPA at day 0. Note that there was a dose-dependent tendency in the proapoptotic effect of VPA on NPCs of ES cell-derived glutamatergic neurons within the range of 0.1–20 mM. Values represent the mean±S.E.M. of three separate experiments

In our culture system, almost all cells are NPCs of ES cell-derived glutamatergic neurons just after thawing and plating (Figure 1a, upper scheme).^33, 34, 37^ The purity of this culture is >97% when measured with immunocytochemistry (ICC) using the glutamatergic neuronal marker vesicular glutamate transporter 1 (VGLUT1) 7 days after plating (Figure 1a, lower panels). To elucidate the effect of VPA on NPCs of glutamatergic neurons at therapeutic levels (0.3–0.7 mM),^4,11^ we treated our NPCs either with 0.5 mM VPA or distilled water (DW; control) immediately after plating (day 0). Remarkably, NPCs treated with VPA died within 24 h, so we investigated caspase-3 activation using ICC to quantify apoptotic neurons 15 h after VPA treatment (Figure 1b). The percentage of cleaved caspase-3-positive neurons significantly increased to 31% (cleaved capase-3^+^ cells per all Tuj1^+^ neurons: 275.7±48.3 per 862.4±101.1 ) in VPA-treated cultures, compared with 19% (cleaved capase-3^+^ cells per all Tuj1^+^ neurons: 231.9±34.2 per 1223.1±157.3 ) in the control (Figures 2a and b). These apoptotic cleaved caspase-3-positive neurons also showed other hallmarks of apoptosis, such as extensive chromatin condensation and nuclear fragmentation, as assessed by Hoechst staining (data not shown). To determine the extent to which the proapoptotic effects of VPA depended on HDAC inhibition,^4, 5^ we also treated NPCs at day 0 with either 100 nM TSA or 5 mM sodium NaB (both HDACis), or 0.5 mM VPM, a structural analog of VPA that lacks HDACi activity. The results were compared with treatment with vehicle controls (dimethyl sulfoxide (DMSO), DW and DMSO). At 15 h after the initiation of treatment with these reagents, we assessed cultures for apoptosis using the methods described above (Figure 1b). Treatment with TSA or NaB dramatically increased the percentage of cleaved caspase-3-positive neurons up to 45% (cleaved capase-3^+^ cells per all Tuj1^+^ neurons: 238.4±7.4 per 562.0±58.5) or 37% (cleaved capase-3^+^ cells per all Tuj1^+^ neurons: 256.7±45.7 per 682.5±103.7), respectively, compared with controls (15% (cleaved capase-3^+^ cells per all Tuj1^+^ neurons: 224.6±38.1 per 1164.4±94.7) and 20% (cleaved capase-3^+^ cells per all Tuj1^+^ neurons: 237.8±34.1 per 1180.7±147.5), respectively; Figures 2a and b). In contrast, VPM did not have any effect on apoptosis (Figures 2a and b). Thus, the proapoptotic effect of VPA on NPCs of glutamatergic neurons was correlated with the inhibition of HDACs. Moreover, to examine the dose-response of VPA on apoptosis of NPCs, we also treated NPCs at day 0 with 0.1–20 mM VPA for 9 h, and investigated caspase-3 activation by ICC (Figure 1b). The proapoptotic effect of VPA on NPCs was observed to be dose dependent within the range of 0.1–20 mM (Figures 2c and d). Proapoptotic effects of TSA, and NaB on NPCs were also dose dependent within the range of 100–300 nM and 1–10 mM, respectively (data not shown). Importantly, the effective dose of VPA in this study (0.5 mM) lies within the therapeutic plasma concentration levels (0.3–0.7 mM),^4,11^ and the doses of other three reagents were also within the range of concentrations common to those of many previous reports.^23, 25, 26, 27, 29, 30^ Thus, all of our remaining studies except for examinations for dose-response of VPA were performed using the concentrations of the four test reagents as mentioned above.

### VPA, TSA, NaB and VPM have no proapoptotic effects on ES cell-derived glutamatergic neurons

**Figure 3:**
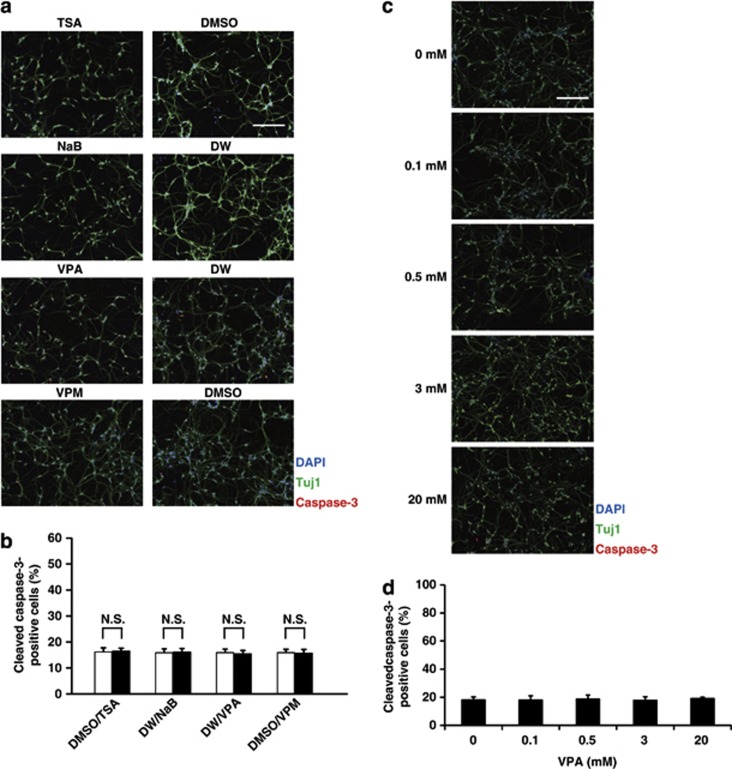
VPA has no proapoptotic effects on ES cell-derived glutamatergic neurons. Glutamatergic neurons were treated with one of the four reagents or their vehicle control at day 3, followed by analysis of caspase-3 activation using ICC 24 h later. They were also treated with different concentrations of VPA at day 3, followed by analysis of caspase-3 activation using ICC 9 h later. (**a**) Representative images of glutamatergic neurons after a 24-h treatment with one of the four reagents or their vehicle control at day 3. Neurons were analyzed by immunofluorescently labeling cells with the neuronal marker Tuj1 (green) and the apoptosis marker cleaved caspase-3 (red). Nuclei were counterstained with DAPI (blue). Scale bar: 200 *μ*m. (**b**) Quantification of the percentage of the cleaved caspase-3-positive neurons (cleaved capase-3^+^ cells among all Tuj1^+^ neurons) after a 24-h exposure to one of the four reagents or their vehicle controls at day 3. Note that none of four reagents had proapoptotic effects on ES cell-derived glutamatergic neurons. Values represent the mean±S.E.M. of seven separate experiments. NS, not significant by one-way ANOVA followed by Tukey–Kramer's *post hoc* test. (**c**) Representative images of glutamatergic neurons after a 9-h treatment with indicated concentrations at day 3. Neurons were analyzed by immunofluorescently labeling cells with the neuronal marker Tuj1 (green) and the apoptosis marker cleaved caspase-3 (red). Nuclei were counterstained with DAPI (blue). Scale bar: 200 *μ*m. (**d**) Quantification of the cleaved caspase-3-positive neurons (cleaved capase-3^+^ cells among all Tuj1^+^ neurons) after a 9-h exposure to VPA at day 3. Note that VPA had no proapoptotic effect on ES cell-derived glutamatergic neurons up to 20 mM. Values represent the mean±S.E.M. of three separate experiments

Almost all of our NPCs are designed to differentiate into glutamatergic neurons within 48 h (Figure 1a, upper scheme).^33, 34, 37^ Therefore, to elucidate the effects of VPA on glutamatergic neurons, we treated the neurons with VPA 3 days after plating (day 3). There were no apparent morphological changes in neurons treated with VPA compared with controls, as assessed by visual inspection. We examined the hallmarks of apoptosis by using the methods described above 24 h after VPA treatment (Figure 1c). ICC analyses revealed that VPA had no proapoptotic effects on these neurons (Figures 3a and b). We also treated the neurons with TSA, NaB or VPM at day 3 and analyzed 24 h later. Compared with the controls, none of the treatment groups showed morphological changes or elicited proapoptotic effects (Figures 3a and b). Furthermore, neurons treated with either of the four reagents remained viable for at least 1 week (data not shown). These data demonstrated that all four reagents had no proapoptotic effects on glutamatergic neurons. To assess the dose-response of VPA on apoptosis of glutamatergic neurons, we also treated neurons at day 3 with 0.1–20 mM VPA for 9 h, and investigated caspase-3 activation by ICC (Figure 1c). VPA had no proapoptotic effect on glutamatergic neurons up to 20 mM (Figures 3c and d).

### VPA, TSA and NaB, but not VPM, rapidly and dramatically enhance the levels of histone H3 acetylation in NPCs

**Figure 4:**
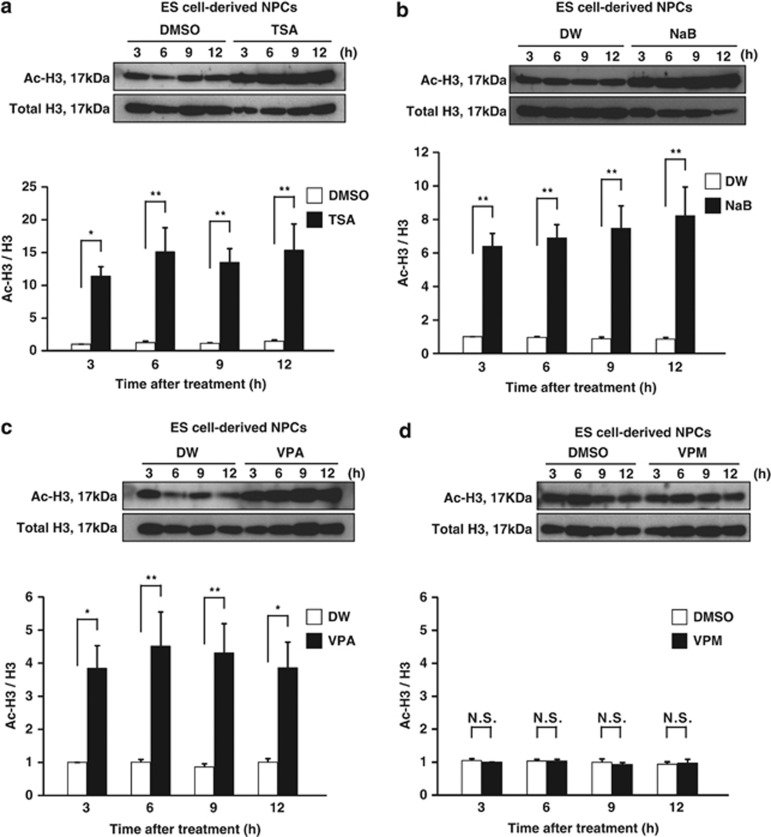
VPA rapidly and dramatically enhances the level of histone H3 acetylation in NPCs. NPCs treated with VPA, TSA, NaB, VPM or their vehicle control at day 0 were subjected to WB analysis to assess histone H3 acetylation 3, 6, 9 and 12 h after treatments. Each panel consists of a typical immunoblot image detected by antibodies against acetylated (upper) and total histone H3 (middle). Quantification of the relative levels of histone H3 acetylation was done using double normalizations (lower): first, by measuring the ratio of acetyl-H3 to total H3 and second by normalizing against the controls 3 h after the treatment. (**a**) Change in the levels of histone H3 acetylation after TSA exposure. (**b**) Change in the levels of histone H3 acetylation after NaB exposure. (**c**) Change in the levels of histone H3 acetylation after VPA exposure. (**d**) Change in the levels of histone H3 acetylation after VPM exposure. Note that VPA, TSA and NaB, but not VPM, rapidly and dramatically increased the levels of histone H3 acetylation in NPCs of ES cell-derived glutamatergic neurons. Values represent the mean±S.E.M. of 10–13 separate experiments. TSA and its control (*n*=10); NaB and its control (*n*=10–12); VPA, VPM and their own controls (*n*=13). *P<0.05; **P<0.01, by one-way ANOVA followed by Tukey-Kramer's *post hoc* test. NS, not significant

**Figure 5:**
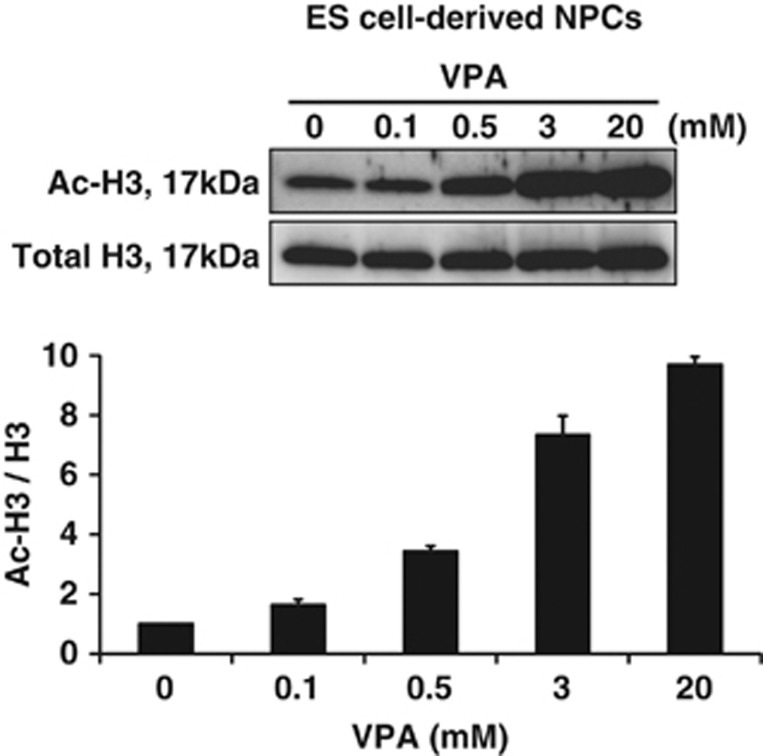
VPA enhances the level of histone H3 acetylation in NPCs in a dose-dependent manner. NPCs treated with different concentrations of VPA at day 0 were subjected to WB analysis to assess histone H3 acetylation after a 9-h exposure. Typical immunoblot images with antibodies against acetylated (upper) and total histone H3 (middle) are shown with quantitative data (lower). The relative levels of histone H3 acetylation was quantified using double normalizations: first, by measuring the ratio of acetyl-H3 to total H3 and second by normalizing against the control. Note that VPA increased the level of histone H3 acetylation in NPCs of ES cell-derived glutamatergic neurons in a dose-dependent manner within the range of 0.1–20 mM. Values represent the mean±S.E.M. of four separate experiments

To investigate the HDACi activity of VPA, TSA and NaB in NPCs of glutamatergic neurons under the experimental conditions described above, we quantified the levels of histone acetylation in NPCs. We treated our NPCs with VPA, TSA, NaB or their vehicle controls at day 0, and harvested cell lysates from each culture 3, 6, 9 and 12 h after the initiation of treatment (Figure 1b). We analyzed cell lysates by western blotting (WB) using antibodies specific for total histone H3 (C-terminus, pan) and acetylated histone H3. The levels of histone H3 acetylation immediately and substantially increased following treatment with VPA, TSA or NaB, and remained elevated for up to 12 h (Figures 4a–c). In contrast, VPM did not affect the levels of histone H3 acetylation (Figure 4d). These data support the notion that the proapoptotic effect of VPA on NPCs of glutamatergic neurons is most likely due to the inhibition of HDACs. To examine the dose-response of VPA on the level of histone acetylation in NPCs, we also treated NPCs at day 0 with 0.1–20 mM VPA for 9 h, and investigated the level of histone H3 acetylation by WB (Figure 1b). VPA enhanced the level of histone H3 acetylation in NPCs of ES cell-derived glutamatergic neurons in a dose-dependent manner within the range of 0.1–20 mM (Figure 5). Taken together with the similar dose-dependent tendency in the proapoptotic effect of VPA on NPCs (Figures 2c and d), these results strongly suggests a correlation between hyperacetylation of histones and apoptosis. Similar increases in histone H4 acetylation were also observed (data not shown), although its quantitative analyses has been hindered by the low sensitivity and specificity of the anti-acetyl histone H4 antibody.

### VPA, TSA and NaB, but not VPM, rapidly increase the levels of histone H3 acetylation in glutamatergic neurons, but less efficiently than in NPCs

**Figure 6:**
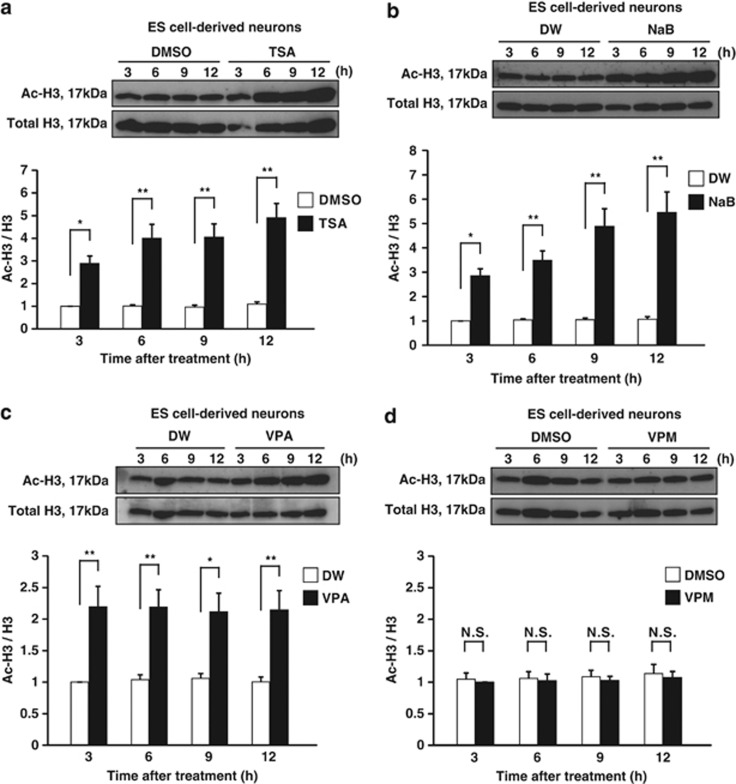
VPA rapidly increases the level of histone H3 acetylation in glutamatergic neurons, but less efficiently than in NPCs. Glutamatergic neurons treated either with VPA, TSA, NaB, VPM or their vehicle controls at day 3 were subjected to WB analysis to determine levels of histone H3 acetylation 3, 6, 9 and 12 h after treatments. Each panel portrays a typical immunoblot image detected by antibodies against acetylated (upper) and total histone H3 (middle). Quantification of the relative levels of histone H3 acetylation was done using double normalizations (lower): first, by measuring the ratio of acetyl-H3 to total H3, and second by normalizing against the controls 3 h after the treatments. (**a**) Change in the levels of histone H3 acetylation after TSA exposure. (**b**) Change in the levels of histone H3 acetylation after NaB exposure. (**c**) Change in the levels of histone H3 acetylation after VPA exposure. (**d**) Change in the levels of histone H3 acetylation after VPM exposure. Note that VPA, TSA and NaB, but not VPM, rapidly enhanced the levels of histone H3 acetylation also in ES cell-derived glutamatergic neurons. Values represent the mean±S.E.M. of 9–11 separate experiments. TSA and its control (*n*=10); NaB and its control (*n*=9–10). VPA, VPM and their own controls (*n*=11). *P<0.05; **P<0.01, by one-way ANOVA followed by Tukey–Kramer's *post hoc* test. NS, not significant

**Figure 7:**
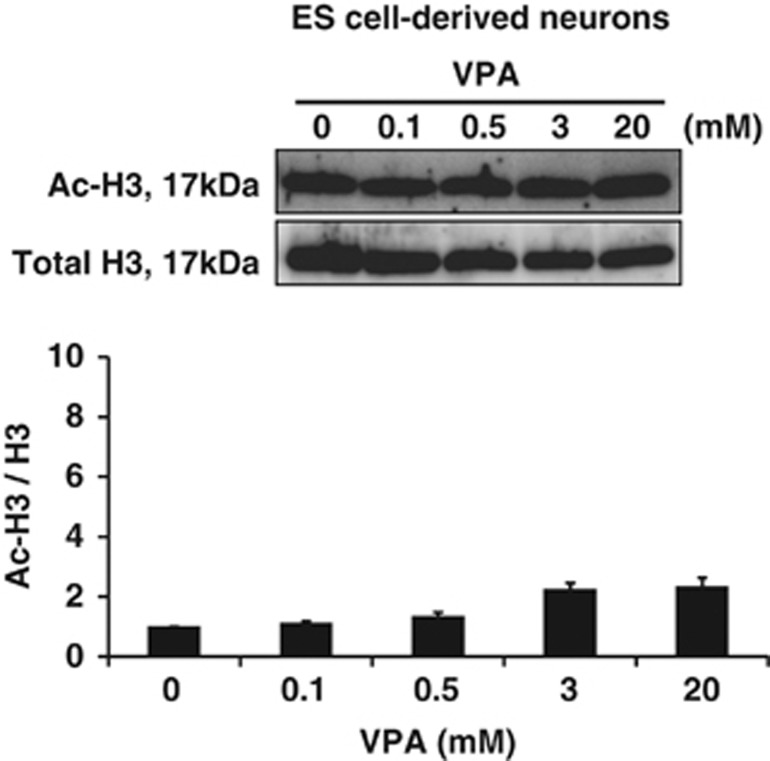
VPA increases the level of histone H3 acetylation in glutamatergic neurons in a dose-dependent manner, but less efficiently than in NPCs. Glutamatergic neurons treated with different concentrations of VPA at day 3 were subjected to WB analysis to determine levels of histone H3 acetylation after a 9-h exposure. Typical immunoblot images with antibodies against acetylated (upper) and total histone H3 (middle) are shown with quantitative data (lower). The relative levels of histone H3 acetylation was quantified using double normalizations: first, by measuring the ratio of acetyl-H3 to total H3, and second by normalizing against the control. Note that VPA enhanced the level of histone H3 acetylation also in ES cell-derived glutamatergic neurons in a dose-dependent manner within the range of 0.1–20 mM, albeit the effect was much weaker than in NPCs. Values represent the mean±S.E.M. of four separate experiments

To examine whether VPA, TSA, NaB and VPM altered the levels of histone H3 acetylation in glutamatergic neurons, we treated our neurons with each of the four reagents at day 3, and harvested cell lysates from each culture 3, 6, 9 and 12 h after the treatments (Figure 1c). WB analyses revealed that the levels of histone H3 acetylation was also enhanced rapidly, but less efficiently than in NPCs, after treatment with VPA, TSA or NaB for up to 12 h (Figures 6a–c). In contrast, VPM did not affect acetylation levels (Figure 6d). These data suggested that VPA also inhibited HDACs in glutamatergic neurons, albeit weakly compared with its effect on NPCs, yet it did not induce apoptosis. To examine the dose-response of VPA on the level of histone acetylation in glutamatergic neurons, we also treated neurons at day 3 with 0.1–20 mM VPA for 9 h, and investigated the level of histone H3 acetylation by WB (Figure 1c). VPA subtly increased the level of histone H3 acetylation also in glutamatergic neurons in a dose-dependent manner within the range of 0.1–20 mM, but the effect was much smaller than in NPCs (Figure 7). Approximately ∼2-fold increase was observed at 20 and 0.1 mM in glutamatergic neurons and in NPCs, respectively (Figures 5 and 7), and apoptosis was not induced under these conditions (Figures 2c, d and ), whereas histone acetylation was increased up to ∼10-fold in NPCs concomitantly with the increase in apoptotic cells (Figures 2c, d and ). Taken together, glutamatergic neurons appear to be more resistant to induction of apoptosis because they are less vulnerable to inhibition of HDACs than their NPCs.

## Discussion

Many previous studies have suggested that VPA has neuroprotective and neurotrophic effects both on NPCs and neurons apart from its function as an AED.^20, 21, 22, 23, 24, 25, 26, 27, 28^ However, contradictory data indicate that VPA and other HDACis may have proapoptotic effects on NPCs and neurons.^29, 30, 31, 32^ This study demonstrated that VPA has no proapoptotic effects on ES cell-derived glutamatergic neurons, but does have proapoptotic effects on their NPCs at therapeutic concentrations (0.3–0.7 mM).^4,11^ Similar phenotypes were induced by treating cells with either of two other HDACis, TSA and NaB, but not with VPM, a structural analog of VPA, which has the same antiepileptic effect as VPA, but does not inhibit HDAC activity. These results suggest that the proapoptotic effect of VPA on NPCs of glutamatergic neurons might result from the inhibition of HDACs. Consistent with this view, we found a robust increase in the levels of histone H3 acetylation in NPCs treated with VPA, TSA or NaB, but not with VPM. The results of dose-dependent effects of VPA both on apoptosis and hyperacetylation of histone H3 in NPCs within the range of 0.1–20 mM (Figures 2c, d and ) also strongly indicates a correlation between hyperacetylation of histones and apoptosis.

Proapoptotic effects of VPA, TSA and NaB on NPCs were dose dependent within the range of 0.1–20 mM, 100–300 nM and 1–10 mM, respectively. Importantly, the effective dose of VPA in this study (0.5 mM) lies within the therapeutic plasma concentration levels (0.3–0.7 mM),^4,11^ and the doses of these reagents were all within the range of concentrations common to those of many previous reports.^23, 25, 26, 27, 29, 30^ In addition to the concentration, duration of exposure to an agent is an important factor for such drug treatments. In contrast to NPCs that rapidly underwent apoptosis, neurons were resistant to VPA treatment for 24 h. Although it was possible that the duration of exposure was too short for neurons, we have also confirmed that neurons treated with either of the four reagents remained viable for at least 1 week.

Many studies have suggested that VPA suppresses apoptosis and induces neuronal differentiation of NPCs through the inhibition of HDACs. Among them, Abematsu *et al.*^27^ specifically examined neurons differentiated from the mouse embryonic forebrain NPCs, which were transplanted into spinal cord injury model mice followed by VPA treatment, and found that 17% were glutamatergic and 70% were GABAergic. Laeng *et al.*^25^ also demonstrated that VPA stimulates GABA neurogenesis from rat forebrain NPCs, but this was not apparently through the inhibition of HDACs. Unlike heterogeneous NPCs isolated from animal brains for primary culture, NPCs used in this study are strongly committed to differentiate into homogeneous glutamatergic neurons, and are most likely induced to undergo apoptosis with VPA through the inhibition of HDACs. These differences can be explained by the action of VPA, which acts primarily to inhibit HDACs, suppress apoptosis, and induce neuronal differentiation of heterogeneous NPCs (especially into GABAergic neurons), but contrarily induces the apoptosis of homogeneous NPCs that are fated to differentiate into glutamatergic neurons.

In this study, VPA, TSA and NaB also increased histone H3 acetylation levels of neurons, albeit weakly compared with their effects on NPCs, but did not significantly affect neuronal survival. Thus, glutamatergic neurons are more resistant to induction of apoptosis because they are less vulnerable to inhibition of HDACs than their NPCs. HDACis are reported to cause acetylated histones to accumulate in tumors (including neuroblastoma cells) as well as in normal tissues (including post-mitotic neurons), but often act selectively to inhibit cell growth of tumors at levels that have little to no toxicity for normal cells.^29, 38^ Laeng *et al.*^25^ also demonstrated that the induction of GABA neurogenesis by VPA treatment was effective only in the undifferentiated population of NPCs. Kataoka *et al.*^32^ showed that proapoptotic and antiproliferative effects of VPA on NPCs in the embryonic neocortex, which might be due to the inhibition of HDACi, was exerted transiently only during early embryonic brain development, especially around E12.5. Thus, the proliferation potentials of cells during HDACi exposure appear to be important for the cellular response.^25, 29, 32, 38^ Our observation of the differentiation stage-specific proapoptotic effect of VPA can be also explained by the division potentials of NPCs and neurons. For further confirmation, we examined the proliferation potentials of our NPCs and neurons by using ICC for the proliferation marker Ki-67, 2 h and 3 days after plating. Expectedly, among the living cells, 80% of the cells after 2 h of plating, which are thought to be mostly still NPCs, were Ki-67-positive, and the percentage decreased to 7% after 3 days, at which point most cells are thought to be neurons (data not shown).

Several studies have proposed that the HDACi action of VPA is closely related to teratogenesis in vertebrate embryos.^4, 18, 19^ However, there are contradictory reports regarding the effects of VPA on apoptotic death of NPCs at developmentally critical periods.^28, 32^ Studies have shown that the reduced (Go *et al.*^28^) or increased (Kataoka *et al.*^32^) apoptotic deaths of NPCs by VPA treatment underlie neurodevelopmental defects. The findings of our study are consistent with those of Kataoka *et al.*

Although further studies are required to clarify the detailed mechanism of how HDAC inhibition causes apoptosis, this is the first report demonstrating the cell type- and differentiation stage-specific proapoptotic effects of VPA on homogeneous NPCs of glutamatergic neurons, which is most likely due to the inhibition of HDACs. Our study implies that the dysfunction of HDACs during pregnancy might be responsible for a high risk of congenital malformation.

## Materials and Methods

### Antibodies and reagents

The nerve-cell culture medium (SBM, Sumitomo Bakelite Co., Ltd., Tokyo, Japan) was used for culture of glutamatergic neurons. All other cell culture reagents have been described previously.^33, 34, 37^ The following reagents were used for experiments investigating the effects of VPA on NPCs and neurons: VPA (Sigma-Aldrich, St. Louis, MO, USA), TSA (Millipore Co., Billerica, MA, USA), NaB (Sigma-Aldrich), VPM (Sigma-Aldrich) and DMSO (Sigma-Aldrich). For ICC, the following antibodies were used: mouse monoclonal antibodies to neuronal class III *β*-tubulin (Tuj1, 1 : 1000; Covance Laboratories, Inc., Berkeley, CA, USA), and Ki-67 (1 : 500; BD Pharmingen, San Diego, CA, USA); rabbit polyclonal antibodies to Tuj1 (1 : 1000; Covance Laboratories, Inc.), VGLUT1 (1 : 1000; Synaptic System, Goettingen, Germany), and cleaved caspase-3 (1 : 200, Cell Signalling Technology, Danvers, MA, USA). Fluorescent mounting medium was purchased from DakoCytomation Inc., Fort Collins, CO, USA. We used the following fluorescence-conjugated secondary antibodies: Alexa Fluor 488- or 568-conjugated goat anti-mouse IgG and goat anti-rabbit IgG (1 : 400; Invitrogen, Carlsbad, CA, USA). For WB, the following antibodies were used: rabbit polyclonal antibodies to histone H3 C-terminus, pan (1 : 50,000; Cat. # 07-690, Millipore Co.) and acetyl-histone H3 (1 : 10,000; Cat. # 06-599, Millipore Co.); and horseradish peroxidase (HRP)-conjugated anti rabbit IgG (1 : 5000; Cell Signaling Technology).

### Cell culture

The ES cell line we selected was E14TG2a (CRL-1821; American Type Culture Collection, Manassas, VA, USA). ES cell-derived glutamatergic neurons were differentiated essentially as we have previously described with a minor modification (see below).^33, 34, 37^ We adjusted the CO_2_ content in the incubators to maintain the pH of the cell culture medium to around 7.4 as previously described.^37^ The temperature of the incubators was kept constant at 37 °C.

All of the dissociated cellular aggregates, which are NPCs, were once frozen. All of our experiments were started from NPCs by thawing and plating them as originally described.^33, 34^ Cells were plated on glass coverslips in 24-well plates at a density of 0.45 × 10^6^ cells per well. Glass coverslips were double coated with poly-𝒟ℒ-ornithine and laminin before use, as originally described.^34^ By changing the medium for glutamatergic neurons from the original medium (complete medium, CM) to a commercially available SBM 48 h after plating, we made a substantial improvement in neuronal survival,^37^ compared with results obtained with a protocol reported by Bibel *et al.*^33, 34, 37^ Although SBM contains glial-conditioned medium, which could theoretically increase the number of non-neural cells, ICC using the glutamatergic neuronal marker VGLUT1 confirmed that this modification did not decrease the purity (>98%).^37^ Purity in our study was even higher than the purity of cultures grown in original CM (90–95%).^33, 34^ Subsequent to this report, we also tried changing to SBM instead of N_2_ medium (N_2_M) 24 h earlier. After confirming that this second modification improved neuronal health and survival without decreasing their high purity (>97% Figure 1a, lower panels), we decided to use SBM from 24 h after plating for all of the following experiments. In all of the experiments investigating the effects of VPA on NPCs and glutamatergic neurons except for those for dose-response of VPA, cells were treated either with 0.5 mM VPA, 100 nM TSA, 5 mM NaB, 0.5 mM VPM, or their vehicle controls either immediately after plating (day 0) or 3 days after plating (day 3). In the experiments for dose-response of VPA, cells were treated with VPA (0.1, 0.5, 3, 20 mM) or its vehicle control instead. The time schedule of fixing cells for ICC and harvesting cells for WB is described in Figures 1b and c. We confirmed that most of our cells were free of mycoplasma, by testing as originally recommended.^34^

### ICC and nuclear staining

Cells cultured on glass coverslips in 24-well plates were washed with phosphate-buffered saline (PBS). After transferring coverslips to a fresh 24-well plates, we fixed cells with 4% paraformaldehyde in 0.1 M phosphate buffer (Wako Pure Chemical Industries, Osaka, Japan) for 10 min. Cells were washed with PBS and incubated for 5 min in permeabilizing solution (PBS containing 0.2% Triton X). After three washes with PBS, cells were incubated for 1 h in blocking solution (PBS containing 5% bovine serum albumin and 0.05% Tween). Subsequently, cells were incubated with primary antibodies for 1 h at room temperature or overnight at 4 °C. After three washes with PBS, cells were incubated with fluorescence-conjugated secondary antibodies for 1 h at room temperature or overnight at 4 °C. After three washes with PBS, nuclei were counterstained with Hoechst 33342 (Invitrogen) or 4′,6-diamidino-2-phenylinodole (DAPI, Dojindo, Kumamoto, Japan). Coverslips were then rinsed three times with PBS and mounted on glass slides. The samples were viewed under an inverted light microscope equipped with epifluorescence and dry condenser for phase-contrast microscopy (DP70, Olympus, Tokyo, Japan) using a 10 × objective.

### Detection of apoptosis

Hallmarks of apoptotic cell death include activation (cleavage) of caspases, condensation, and fragmentation of nuclei and formation of apoptotic bodies. We investigated caspase-3 activation by using ICC and examined extensive chromatin condensation and nuclear fragmentation by using Hoechst staining. Neurons were analyzed by immunofluorescence labeling with the neuronal marker Tuj1 (green) and the apoptotic marker cleaved caspase-3 (red), and nuclei were counterstained with Hoechst 33342 or DAPI (blue). We randomly obtained four representative images per well under the microscope with a 10 × objective, and counted all of the cells in those images for one experiment. We quantified the percentage of the cleaved caspase-3-positive neurons (cleaved caspase-3^+^ cells among all Tuj1^+^ neurons) in each culture. More than 200 cells were counted in each of at least three independent experiments in order to quantify cleaved caspase-3-positive cells.

### Analysis of histone acetylation levels by WB

NPCs or glutamatergic neurons were treated with VPA, TSA, NaB, VPM or their vehicle controls, and harvested 3, 6, 9 and 12 h after the treatment (Figures 1b and c). Whole-cell lysates were prepared by boiling samples in SDS-PAGE sample buffer for 5 min, separated by SDS-PAGE, and transferred electrophoretically onto polyvinylidene difluoride membranes (Millipore Co.). The blotted membranes were blocked for 1 h with 5% non-fat dry milk in PBS containing 0.05% Tween-20 (PBS-T) and incubated for 2 h at room temperature or overnight at 4 °C, with the primary antibody against anti-acetyl-histone H3 diluted in PBS-T containing 1% non-fat dry milk. After washing in PBS-T, the membranes were incubated for 1 h at room temperature or overnight at 4 °C, with an HRP-conjugated anti-rabbit IgG antibody (1 : 5000). The immune complexes were visualized using the ECL chemiluminescence system (GE Healthcare, Buckinghamshire, UK) with the LAS-3000 image analyzer (Fuji Film, Tokyo, Japan). Then, the membranes were stripped for 30 min at 37 °C in Restore Plus Western Blot Stripping Buffer (Thermo Scientific, Rockford, IL, USA) and reprobed with anti-histone H3 and HRP-conjugated anti-rabbit IgG antibodies as above. Note that no signal was detected when the second primary antibody (anti-histone H3) was added in the second blotting, indicating that both the first primary and secondary antibodies were efficiently stripped off from the membrane. We measured the intensity of acetyl-histone H3 and total signals using Multi Gauge (Fuji Film), and the acetylation level in each time point was expressed as the relative value to each vehicle control at 3 h after the treatment by double normalization; first by measuring the ratio of acetyl-H3 to total H3 and secondly by normalizing against the control. We performed 9–13 independent experiments for quantification. For Figures 5 and 7, the acetylation level in each VPA concentration was expressed as the relative value to vehicle control by double normalization; first by measuring the ratio of acetyl-H3 to total H3 and secondly by normalizing against the control. Although we also tried to perform similar analyses for histone H4 acetylation, we experienced difficulty with quantitative analyses because of the poor sensitivity and specificity of the anti-acetyl histone H4 antibody.

### Statistical analysis

The quantitative data are expressed as mean±S.E.M. of at least three (indicated in the figure legends, when the number was >3) independent experiments. All statistical analyses of these values were performed using one-way ANOVA followed by Tukey–Kramer's *post hoc* test. Values of *P*<0.05 were considered statistically significant.

## Abbreviations

ES cell: embryonic stem cell
NPC: neural progenitor cell
VPA: valproic acid
TSA: trichostatin A
NaB: sodium butyrate
VPM: valpromide
DW: distilled water
DMSO: dimethyl sulfoxide
AED: antiepileptic drug
GABA: *γ*-aminobutyric acid
HDAC: histone deacetylase
HDACi: HDAC inhibitor
ERK: extracellular regulated kinase
PKC: protein kinase C
GSK-3*β*: glycogen synthase kinase-3*β*
N_2_M: N_2_ medium
CM: complete medium
SBM: the nerve-cell culture medium from Sumitomo Bakelite Co., Ltd.
ICC: immunocytochemistry
WB: western blotting
Tuj1: neuronal class III *β*-tubulin
VGLUT1: vesicular glutamate transporter 1
DAPI: 4′,6-diamidino-2-phenylinodole
PBS: phosphate-buffered saline
PBS-T: PBS containing 0.05% Tween-20
